# O23 - Functional effects of an amino-acid based formula with synbiotics in cow’s milk allergic infants

**DOI:** 10.1186/2045-7022-4-S1-O23

**Published:** 2014-03-14

**Authors:** Wesley A Burks, Lucien F Harthoorn, Jane E Langford, Marleen T Van Ampting, Steven B Goldberg, Peck Y Ong, Brandon J Essink, Robert B Scott, Bryan M Harvey

**Affiliations:** 1 University of North Carolina, Chapel Hill, NC, USA; 2 Nutricia Advanced Medical Nutrition, Danone Research – Centre for Specialised Nutrition, Liverpool, United Kingdom; 3 Visions Clinical Research, Tucson, AZ, USA; 4 Children’s Hospital Los Angeles, Los Angeles, CA, USA; 5 Meridian Clinical Research, Omaha, NE, USA; 6 Asheboro Research Associates, Asheboro, NC, USA; 7 Children’s Investigational Research Program, LLC (CHIRP), Bentonville, AR, USA

## 

Pre- and probiotics (synbiotics) are suggested to have beneficial effects on human health. This study describes effects of an amino acid based formula (AAF) with synbiotics in infants with cow’s milk allergy (CMA).

In a prospective, randomized, double-blind controlled study, full term infants with diagnosed IgE and/or non-IgE mediated CMA received a commercially available AAF (NEO; n=56) or an AAF with synbiotics (neutral and acidic oligosaccharides, *Bifidobacterium breve* M-16V) (NEO-SYN; n=54) for 16 weeks. Primary outcome was growth and formula tolerance; secondary outcomes were dermatological/respiratory allergic characteristics, and stool characteristics recorded in subject diaries and evaluated by a physician. Furthermore, adverse events and concomitant medications used during the study were reported.

Average age of infants at inclusion was 4.5±2.4 months. Overall, NEO-SYN and NEO were equally tolerated and both supported normal growth. Both formulas reduced allergic symptoms; SCORAD decreased in both groups with no significant differences in SCORAD change over 16 weeks between both groups. Softer and yellow/brown stools were reported more frequently in the NEO-SYN group compared to more dry and green/dark brown stools in the NEO group (week 0-2, p=0.035; week 2-4, p=0.010 for stool consistency). Furthermore, the NEO-SYN group had less subjects with reported infections (p=0.008) and less subjects receiving medication for functional gastrointestinal disorders (p=0.029) when compared with the NEO group. In addition the NEO-SYN group had a lower number of infants on antibiotics (p=0.049), especially amoxicillin (p=0.004).

This study shows that an AAF with synbiotics is equally tolerated, supports normal growth and is similarly effective in the management of CMA symptoms compared to an AAF without synbiotics. Infants taking an AAF with this synbiotic blend have different stool characteristics, less reported infections and less antibiotic use, which suggests that addition of the synbiotics to an AAF improves resistance to infections and reduces specific medication use.

